# Epidemiological pattern of rape cases managed at a regional hospital in South Africa

**DOI:** 10.4102/hsag.v29i0.2434

**Published:** 2024-01-15

**Authors:** Chika K. Egenasi, Mathew A. Benedict, Anthonio O. Adefuye, Lynda U. Madu

**Affiliations:** 1 Department of Family Medicine, Faculty of Health Sciences, University of the Free State, Bloemfontein, South Africa; 2 Department of Basic Medical Sciences, College of Dental Medicine, Kansas City University, Joplin, Missouri, United States of America; 3 Department of Health Sciences Education, Faculty Health Sciences, University of the Free State, Bloemfontein, South Africa; 4 Department Microbial, Biochemical and Food Biotechnology, Faculty of Natural and Agricultural Sciences, University of the Free State, Bloemfontein, South Africa

**Keywords:** rape, sexual assault, South Africa, epidemiological pattern, Robert Mangaliso Sobukwe Hospital

## Abstract

**Background:**

South Africa has one of the highest incidences of rape globally. Understanding the epidemiological pattern of rape is needed to inform the design of effective intervention programmes for rape prevention and management of alleged rape cases.

**Aims:**

To investigate important epidemiological patterns associated with rape in Kimberly, Northern Cape Province, South Africa.

**Setting:**

The Robert Mangaliso Sobukwe Hospital (RMSH) forensic unit.

**Methods:**

A descriptive, retrospective cross-sectional clinical audit of rape cases.

**Results:**

The majority (93.3%) of the alleged rape victims were women, with a mean age (SD) of 21.6 years (11.3); the male population made up 6.7% of the cases, with a mean age (SD) of 10.5 years (6.9). The highest incidence of alleged rape in the male population was seen in the age group ≤ 16 years (81.8%) and for women 17–30 years (50.3%). Most of the incidents occurred at the perpetrators’ homes (42.7%); on the days Fridays (14.6%), Saturdays (29.9%) and Sundays (23.2%); at night up to midnight 20:00–23:59 (32.9%) (*p* = 0.01) and involved threats of violence (55.5%). The majority (56.0%) of the perpetrators were known to the victims.

**Conclusion:**

Important information about the victims and circumstances in which rape occurs as reported herein can be used to inform the design of effective intervention programmes for sexual crime prevention and management in Kimberly, South Africa.

**Contribution:**

This study helped to advance knowledge and understanding of the epidemiological pattern associated with rape in Kimberley, Northern Cape Province of South Africa.

## Introduction

Rape is an invasive violation of the integrity of a person’s body; it is one of the most devastating personal traumas and can lead to loss of life and invasion of psychological and physical privacy (Naidoo 2013). Rape is described as the penetration of the mouth, vagina or anus by any part of an attacker’s body or by an object used by the attacker, without the victim’s consent (Easteal 1992). South Africa has one of the highest incidences of rape globally and has been called the rape capital of the world (Naidoo 2013). The reported rape rate in South Africa was 72.1/100 000 in 2019/2020 (World Population Review 2023). According to the South African Police Service (SAPS) crime statistics report, 41 739 rape cases were recorded in South Africa between April 2021 and March 2022 (South African Police Service 2022).

Most cases of rape in South Africa go unreported, because of a lack of faith in the criminal justice system and medical services and fear of secondary trauma (Naidoo 2013). Victims of rape are often women and children, with very few rape cases involving men being reported because of fear of stigma and prevailing gender stereotypes (Jina et al. 2020). Harmful cultural practices and beliefs that undermine the rights of women and children have been implicated in promoting a rape culture in South Africa. Cultural practices, such as ‘*Ukuthwala*’ – a practice that involves a man abducting a young girl and forcing her into marriage, predominantly practiced among Xhosa-speaking tribes in South Africa – have been implicated as an enabler for rape and sexual violence (Machaka 2018). Other factors include the high rate of unemployment, social inequality and patriarchal beliefs (Finchilescu & Dugard 2021). A range of measures intended to combat the incidence of rape and improve the management of rape survivors has been introduced by the South African government over the years (Vetten 2011). Despite these measures, rape prevention remains ineffective, and successful conviction rates for rape crimes remain low (Fouche et al. 2018).

Understanding of the epidemiological pattern (i.e. the who, when, where and how) of rape is needed to inform the design of effective intervention programmes for rape prevention and management of alleged rape cases (Swart et al. 2000). The Robert Mangaliso Sobukwe Hospital (RMSH) forensic unit is one of the dedicated referral centres for alleged rape cases in the Northern Cape Province, South Africa. Using a retrospective cross-sectional clinical audit approach, this study reviewed all alleged rape cases managed at the RMSH forensic unit in a 2-year period (January 2020–December 2021), in order to establish epidemiological patterns that can be used in intervention programmes for rape prevention.

## Methods

### Study design

This was a descriptive, retrospective cross-sectional clinical audit of all alleged rape cases managed at RMSH forensic unit from 01 January 2020 to 31 December 2021.

### Study setting

Kimberley, the capital of the Northern Cape Province of South Africa, has a landmass density of about 372 889 square kilometres, most of which is rural. According to the 2011 census figures, Kimberley had a population of 225 200 (Statistics SA 2011). Kimberley Hospital, now RMSH, is a regional and tertiary hospital in the Northern Cape; it is the only referral centre in the Northern Cape and receives referred patients from all over the province. Similarly, the RMSH forensic department is a referral centre for adult victims of rape and children younger than ≤ 13 years in the province.

### Data collection

Particulars of all alleged rape cases managed at the RMSH forensic department from 01 January 2020 to 31 December 2021 were retrieved from the patient register in the forensic department, and patient statistics records kept by the head of the forensic department. The applicable case records were subsequently retrieved from the records department. A datasheet designed by one of the researchers, based on trends observed in similar a study, was used to collect data from the patients’ clinical notes and the J88 form (medico-legal report) (Kotzé, Brits & Botes 2014). The datasheet was pre-tested on the first 20 files (in succession). This ensured that the variables on the datasheet are well and correctly structured. It also ensured that data were accurately captured and quality maintained. No significant changes resulted from the pre-testing, and the data obtained from the 20 patient files were included in the overall data of the study.

#### Inclusion criteria

- Positive history of rape regardless of age,
- Patient brought in by a law enforcement officer, who had to have completed the SAPS 308 Form (Authorisation form) (Kotzé et al. 2014) and
- Patient examined and managed at the RMSH forensic unit and had to have a J88 form duly completed (Kotzé et al. 2014).

#### Exclusion criteria

Patients with no evidence collected, incomplete medical records and no J88 form completed.

### Data analysis

Data were entered into an Excel spreadsheet (Microsoft Office Professional Plus 2016) and analysed using the Statistical Package for Social Sciences (IBM SPSS Statistics 25). Results are presented in contingency tables as frequencies and percentages. Chi-squared test and Cramér’s V were used to examine the existence and the strength of an association between cross-tabulated variables.

### Ethical consideration

The Northern Cape Department of Health approved the protocol for the study with approval no. NC_2022RMSH04_001. No patient names or personal identifiers appear in the data collection forms. All patient records were kept in a secure location and were only available to only one of the researchers (CE), who collated the data. All patient information and records were managed in a strictly professional and confidential manner.

## Results

Only 164 of the 172 case files reviewed were included in this study. The remaining 8 patient files had incomplete information and could not be analysed. Less than half (32.3%; *n* = 53) of the cases were reported in the year 2020, while the majority (67.7%; *n* = 111) were reported in 2021.

### About the victim (Who)

#### Demographic profile

The majority of the alleged rape victims were female (153; 93.3%) with mean age (SD) of 21.6 years (11.3), while male victims accounted for only 6.7% (*n* = 11) of cases, with a mean age (SD) of 10.5 years (6.9). The highest incidence of alleged rape cases in male and female victims was seen in the age groups ≤ 16 years (*n* = 9; 81.8%) and 17–30 years (*n* = 77; 50.3%), respectively (Table 1). The races of these patients were deliberately not recorded in the case files. This was done to avoid racial profiling.

**TABLE 1:** Age distribution of alleged rape victims.

Gender	Age group (years)										Total	
	≤ 16		17–30		31–50		51–70		> 71			
	*n*	%	*n*	%	*n*	%	*n*	%	*n*	%	*n*	%
Female	53	34.6	77	50.3	19	12.4	2	1.3	2	1.3	153	100.0
Male	9	81.8	1	9.1	0	0.0	0	0.0	1	9.1	11	100.0

**Total (*n*)**											**164**	

#### Mental status of the victims

Although the majority (*n* = 155, 94.5%) of the alleged rape victims were mentally competent, 5.5% (*n* = 9) were recorded to be mentally incompetent.

#### Use of recreational substances

Only 39 (23.8%) of the victims reported to have ingested alcohol or spiked drinks. No other recreational substances were reported.

#### Comorbidities (HIV)

Only 26.2% (*n* = 43) of the victims knew their HIV serostatus, while the remainder (*n* = 121, 73.8%) reported not knowing their HIV status. The majority (*n* = 41; 95.3%) of those who reported knowing their HIV serostatus willingly disclosed this information to the healthcare provider. About half (*n* = 22, 51.2%) of those who disclosed, reported to be HIV seropositive [χ^2^ = 148.9, Cramér’s *V* = 0.95; *p* = 0.01].

### Circumstances in which the rape occurred (Where, When and How)

#### Day and time of incidence

**FIGURE 1:**
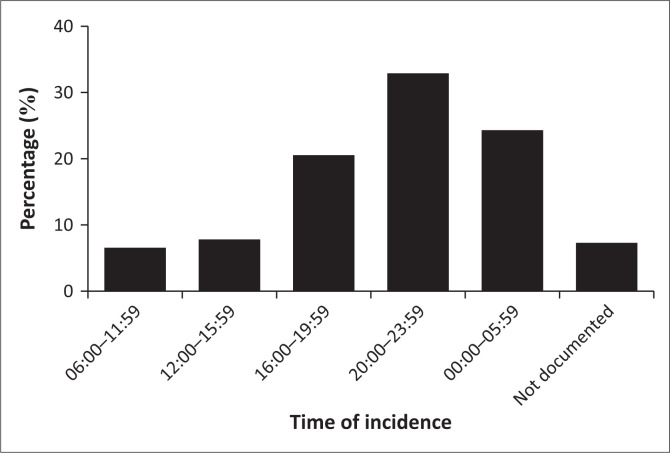
Time of incidence of alleged rapes.

Most of the incidences occurred on the weekend days of Fridays (*n* = 24, 14.6%), Saturdays (*n* = 49; 29.9%) and Sundays (*n* = 38; 23.2%), at night up to midnight (20:00 23:59) (*n* = 54; 32.9%) [χ^2^ = 71.3, Cramér’s *V* = 0.29; *p* = 0.01] (Figure 1).

#### Place of occurrence

**FIGURE 2:**
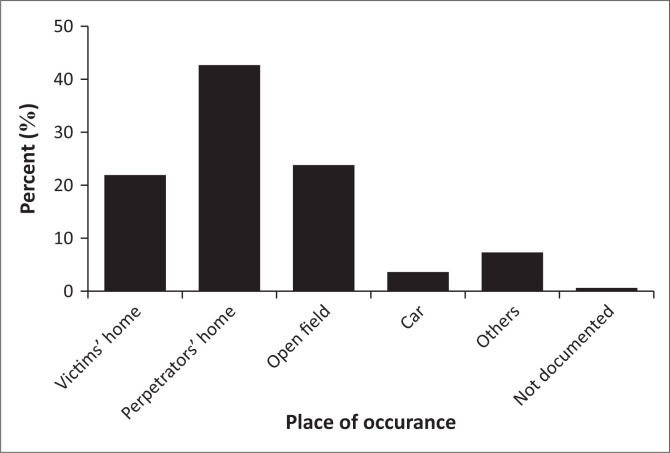
Place of occurrence of alleged rape.

Most of the incidences were reported to have occurred at the perpetrators’ homes (*n* = 70; 42.7%) or on an open field (*n* = 39; 23.8%), while only 22.0% (*n* = 36) took place at the victims’ homes (Figure 2).

#### Associated threat of violence

The majority (*n* = 91; 55.5%) of the cases involved the threat of violence. More than half (*n* = 86; 56.2%) of the female victims reported being subjected to threats of violence, while only 45.5% (*n* = 5) of the male victims reported this [χ^2^ = 0.4, Cramér’s *V* = 0.05; *p* = 0.4]. Physical bodily assault and victim abduction were recorded in only 17.1% (*n* = 28) and 12.8% (*n* = 21) of cases, respectively.

#### Visible evidence of penetration

Visible evidence of vaginal and anal penetration was documented in 84 (51.2%) and 10 (6.1%) cases, respectively. Anal penetration was documented for 4 (2.6%) and 6 (54.5%) female and male victims, respectively [χ^2^ = 48.3, Cramér’s *V* = 0.5; *p* = 0.01].

#### Use of protection

About half (*n* = 86, 52.4%) of the victims reported that the perpetrator did not use any form of protection (condom), 15.9% (*n* = 26) were unsure, while only 11.6% (*n* = 19) reported that a form of protection (condom) was used during the alleged act of rape. This information was not documented for 33 (20.1%) cases. It is interesting that the majority (*n* = 13, 59.1%) of those who reported to be HIV positive were raped without protection (Table 2).

**TABLE 2:** The use of protection during rape.

HIV status	Was a condom used?								Total	
	Yes		No		Not sure		Not documented			
	*n*	%	*n*	%	*n*	%	*n*	%	*n*	%
HIV seropositive	2	9.1	13	59.1	6	27.3	1	4.5	22	100.0

#### Perpetrators

The majority (*n* = 92; 56.0%) of the alleged perpetrators were known to the victims, and in 73.9% (*n* = 68) of cases, the perpetrators were friends of the victim (Table 3). The majority (*n* = 146, 89.0%) of incidents involved only one perpetrator, while ≥ 2 perpetrators were reported in 11.0% (*n* = 18) of cases.

**TABLE 3:** Identity of alleged perpetrators of rape.

Known perpetrator	Identity of perpetrator																Total	
	Stepfather		Uncle		Father		Friend(s)		Other relatives		Neighbour		Others		Not documented			
	*n*	%	*n*	%	*n*	%	*n*	%	*n*	%	*n*	%	*n*	%	*n*	%	*n*	%
Yes	1	1.1	3	3.3	1	1.1	68	73.9	5	5.4	6	6.5	7	7.6	1	1.1	92	100.0
No	0	0.0	0	0.0	0	0.0	0	0.0	0	0.0	0	0.0	0	0.0	72	100.0	72	100.0

**Total (*n*)**																	**164**	

### Post-rape care

#### Time of presentation

The majority (*n* = 145; 88.4%) of the victims presented at the hospital within 72 h of the alleged incident, while only 3.0% (*n* = 5) and 4.9% (*n* = 8) presented ≥ 72 h and ≥ 7 days, respectively. All the victims were brought to the hospital by a law enforcement officer (member of the SAPS) and were attended to by a healthcare provider trained in clinical forensic medicine.

#### Medical care

HIV postexposure prophylaxis (PEP) was administered and documented in 121 (73.8%) cases, not documented in 12 (7.3%) cases and not administered in 31 (18.9%) cases. Further analysis reveals that 71.0% (*n* = 22) of those who were not given PEP had already reported HIV seropositive status and were on medication. The majority (*n* = 157; 95.7%) of the cases were referred to Thuthuzela Care Centre for rape counselling.

## Discussion

Gender-based violence (GBV) is a common topic of discussion in South Africa, as it is a profound and widespread problem that affects many aspects of society (Oparinde & Matsha 2021). Adolescent women are often the victims of rape and sexual assault although it may affect people of all ages and genders (Fakunmoju et al. 2021). This study confirmed this claim, as the overwhelming majority of the victims in this study were women between the ages of 17 years and 30 years. This phenomenon has been attributed to cultural practices and traditions that promote gender inequality and discriminate against women (Ngidi, Moletsane & Essack 2021). In addition, the patriarchal nature of the African society and, indeed, South Africa, promotes the notion that men own their female partners and have the right to have sex with them whenever the men desire, which means women have little or no control over their bodies and sexualities (Sikweyiya et al. 2020). In a study by Jewkes et al., rape perpetrators indicated that their motivation to rape emanated from a sense of entitlement over their victims (Jewkes et al. 2010). The perception that sexual abuse of boys is a rare or even non-existent public health problem has changed substantially in recent years. In a longitudinal study aimed at investigating the perspective on boys as victims of childhood sexual abuse in South Africa, Richter et al. report that South African boys experience high levels of sexual abuse (Richter et al. 2018). Similarly, findings from this study reveal that the majority of male victims of rape were boys ≤ 16 years of age. It has been reported that boys who experience childhood sexual abuse tend to be smaller (shorter) and are from poorer families (Richter et al. 2018). Although this study did not investigate the stature or the socioeconomic status of victims, it is very plausible that these young boys fall within the aforementioned categories.

Fewer incidents of rape were reported in 2020 than in 2021. This could be because of the hard lockdown implemented by the South African government at the peak of the coronavirus disease 2019 (COVID-19) pandemic in 2020. Lockdown regulations, such as a ban on alcohol sales, were reported to have positive effects on health services in South Africa and included a 91.6% reduction in sexual assault cases (De Jong et al. 2020). However, some studies also reported an increase in gender-based domestic violence during the COVID-19 lockdown, as restrictions on movement meant that women at risk of domestic violence were confined to their homes with their abusive partners (Nduna & Tshona 2021). Easing the lockdown restrictions and regulations may account for the increase in the number of alleged rape incidents recorded in 2021 by this study.

The findings in this study show a strong and significant association between the incidence of alleged rape and when it occurs, namely mostly on weekend nights, up to midnight. This finding corroborates that of Swart et al. (2000), who report that the incidence of rape in Johannesburg, South Africa, was found to dramatically increase from Fridays, with the highest incidence on Saturday, followed by Sundays (Swart et al. 2000). In addition, they report that most rapes reported in the Johannesburg area occurred between 18:01 and 00:00 (Swart et al. 2000). This suggests a link between the incidence of rape and increased social gatherings that often take place, sometimes up to midnight, on the weekend days in most parts of South Africa. Most rapes are reported to occur at social gatherings while people are consuming alcohol (Cole 2006). In South Africa, a person cannot legally consent to sex if they are intoxicated (South African Government 2007). In this study, only a small number of victims of rape reported having consumed alcoholic beverages. This is contradictory to the findings of Oshodi et al. in a Cape Town study, where 58% of the rape victims reported having used alcohol (Oshodi et al. 2020). This suggests that one can be a victim of sexual assault regardless of the level of alcohol intoxication. While the majority of the victims were mentally competent, about 5.5% (*n* = 9) were recorded to be mentally incompetent and may have been incapable of consenting to sexual acts. This corroborates findings from studies that report that individuals with mental retardation are increasingly becoming victims of sexual abuse, including rape, in South Africa (Calitz 2011). The ingrained reliance of such people on the caregiver authority figure, emotional and social insecurities, ignorance of sexuality and sexual abuse and a powerless position in society have been documented as some of the reasons why mentally incompetent individuals are especially prone to sexual abuse (Morano 2001). Because the vast majority of these individuals are often unable to testify in court, the majority of perpetrators tend to avoid arrest, because of lack of evidence (Kheswa 2014). However, Pillay and Sargent (2000) advocate that a non-intimidatory approach, characterised by developmentally sensitive interviewing and the use of an intermediary system can substantially increase the individual’s level of confidence and enable them to relate the events in simple terms and give credible evidence in court (Pillay & Sargent 2000). It has been reported that primary care physicians (PCPs) have a potentially powerful role in preventing and documenting cases of sexual abuse of people who are affected by intellectual disabilities in their communities (Morano 2001). During clinical visits, PCPs must look out for signs of sexual abuse or sexual activity and must exhibit courage and report any irregularities (Morano 2001). In addition, PCPs should be aware that people who are mentally incompetent and who live in community group homes can be easy targets for abuse by caregivers or other members of the community as the majority of abusers are known to these individuals (Morano 2001).

The findings in this study reveal that more than half the cases also involved threats of violence. Some of the victims reported being physically, bodily assaulted and abducted by the alleged perpetrators. This suggests high rates of violence in sexual assault scenarios, which highlights the need for comprehensive improvements to social policy and hospital emergency and clinical forensic services, as the proper, unambiguous description and interpretation of injuries to areas other than the genitals might be crucial and important in corroborating a victim’s account of events (Crane 2013). The majority of the incidents of alleged rape reported by victims in this study took place at the homes of the perpetrators of the rape (42.7%), in open fields in the community (23.8%) or at the victim’s home (22.0%). This is similar to findings by Swart et al., who report that a significant number of rape incidents occur at a perpetrator’s home, in an open field and at the victim’s home (Swart et al. 2000). While open spaces in urban areas are critically important for ensuring the continued presence of nature and related natural resources in built environments, these areas are often regarded as crime hotspots and refuge areas for potential criminals (Perry, Moodley & Bob 2008). It has been reported that the greatest proportion of gang rapes in South Africa occur in public spaces, such as open spaces and parks, stretches of veld and parking areas (Vetten & Haffejee 2005). This suggests that there is a need to introduce innovative safety measures to secure women’s freedom of movement. Increasing security and lighting in parks at night and over weekends; carrying out targeted police patrolling, particularly at night and over weekends, in areas surrounding public spaces; and the installation of CCTV cameras in parking areas and parks have been suggested as innovative measures that can help curb rape in open spaces (Vetten & Haffejee 2005). All the rape survivors in this study were brought to the hospital by a member of the SAPS Family Violence, Child Protection and Sexual Offences (FCS) unit. Members of the FCS unit ensure that survivors are treated with the utmost care to avoid secondary victimisation and to investigate the cases (Geldenhuys 2019). Their services have assisted in the successful prosecution of perpetrators of sexual offences (South African Police Service 2021). It has been reported that medico-legal assessment, including the physical examination and evidence collection in a rape case, should never be delayed (Kotzé & Brits 2017). Freedman (2020) reports that a forensic examination must take place within 7 days of the sexual assault (Freedman 2020). Delayed presentation may unfavourably impact medical treatment and affect the quality of evidence and case outcomes, since the probability of recovering foreign DNA diminishes with loss of foreign body fluid (Klemmer, Neill & Jarvis 2021). The findings in this study reveal that a minority (4.9%) of the victims presented ≥ 7 days after the assault. In these cases, finding credible forensic evidence might be unlikely, and it may be difficult to successfully prosecute the alleged perpetrators. In addition, these women are unlikely to have received the appropriate medical care, thus increasing their risk of contracting debilitating illnesses, such as sexually transmitted infections and HIV. Factors such as internal psychological barriers (e.g. shame, anxiety and fear) or environmental factors (e.g. prior relationship with perpetrator) have been reported as barriers to early reporting of rape (Jones et al. 2009). It is, therefore, plausible that women who report late were affected by such psychological and environmental factors. All victims of rape in this study were seen by doctors trained in clinical forensic medicine and working in the RMSH forensic unit. It is paramount that rape victims be seen by healthcare practitioners trained in forensics, to ensure proper medico-legal documentation of evidence and to ensure that the chain of custody is preserved and admissible in a court of law (Pitre & Lingam 2013).

The findings in this study reveal that the majority of the perpetrators were known to the victims; they were usually friends of the victim. This is in keeping with findings of prior studies that report that perpetrators of rape were, in most cases, known to the victims (Ngubane et al. 2022; Parcesepe et al. 2016). Findings of this present study, namely that most incidents of rape were by a single perpetrator, confirm a report by Swart et al. (2000). The authors found that rapes committed by ≥ 2 persons account for only 11.0% of cases, which corresponds with the national average reported by Statistics South Africa in their report, titled *Quantitative Research Findings on Rape in South Africa* (Hirschowitz, Worku & Orkin 2000). Vetten and Haffejee report that incidents of gang rape are often predatory, daring and violent (Vetten & Haffejee 2005). Similarly, Ullman reports that victims of gang rape fared much worse and received more negative social reactions from people they told about their assaults, than victims who were raped by a single person (Ullman 2007).

When examining a person claiming to have been raped, genital injuries are the first to come to mind; however, these injuries are the hardest to find, because they can be minimal and may heal rapidly (Gomes 2019). It is important to document genital injuries, because they help prove whether penetration occurred (Gomes 2019). In this study, visible evidence of anal and vaginal penetration was documented for most of the victims examined. This finding is supported by reports from studies that emphasise the importance of genital examination and proper documentation of genital injuries of rape victims (Basile et al. 2021; Kotzé & Brits 2017). It should be noted that the absence of genital injuries does not indicate that there was consent (Larkin et al. 2012). Nonreporting of genital injuries may be because of the difficulty in finding small injuries that may have been missed or may have healed (Gomes 2019). Various studies report greater success in noticing injuries if staining techniques are used, such as toluidine blue combined with colposcopy (Jones et al. 2003; Larkin et al. 2012). In this study, anal injuries were mostly documented in male victims (54.5%), similar to findings by Manchisa et al., who report anal injuries in 57.8% of male victims of rape in their study (Machisa et al. 2018).

In this study, few victims reported that condoms had been used by perpetrators; most of the perpetrators did not use condoms, and in certain instances, the victims were unsure if condoms were used. While the HIV status of most of the rape victims in this study was unknown, a more worrying finding is that the majority (59.1%) of those who reported to be HIV seropositive were raped without the use of condoms. Corroborating studies report that the high prevalence of rape in South Africa fuels the AIDS epidemic (Armstrong 1993; Bello & Pather 2008). In addition to the possibility of HIV being transmitted, the incident of rape can increase the risk of contracting other sexually transmitted infections, such as chlamydia, herpes and gonorrhoea and becoming pregnant (Jewkes et al. 2009). The authors found that HIV PEP, to prevent the transmission of HIV, was administered and documented in the majority (73.8%) of cases. This finding confirms studies that report on the benefits of administering PEP to victims of rape (Kim, Martin & Denny 2003; Meel 2005). The consequences of not giving HIV PEP may be life-altering for victims, hence the need to ensure that it is given when required (Draughon 2012). This study did not look at adherence to PEP. Chacko et al. in their systemic review and meta-analysis of PEP in victims of sexual assault, report that adherence to PEP was poor in all settings (Chacko et al. 2012). It is, therefore, plausible that some of the victims in this study did not adhere to PEP.

Most of the rape survivors in this study were referred to the Thutuzela Care Centre for multidisciplinary post-rape care and counselling to prevent the progression to post-traumatic stress disorder and other complications that could follow trauma associated with rape (Sepeng & Makhado 2019). Future research on sexual abuse should be focused on understanding the perpetrators of rape in Kimberley and why they do it.

## Strength and limitations of the study

The main strength of this study is that the important information about the victims (Who), circumstances in which the rape occurred (Where, When and How), and post-rape care given presented herein can be used to inform the design of effective intervention programmes for sexual crime prevention and management in Kimberly, Northern Cape Province of South Africa. With respect to limitations, the study was limited by its focus on victims of rape in Kimberley only, and the findings may differ from what is obtainable elsewhere in South Africa and other countries; hence, the results cannot be generalised.

## Conclusion

Rape is a violent act that leaves victims mentally and physically traumatised. This study focused on rape victims who attended the RMSH, Kimberley and has helped to advance knowledge and understanding of the epidemiological pattern associated with the subject in Kimberley, the capital city of the Northern Cape Province of South Africa. This study corroborates the findings of other studies on this subject.
